# Functional Properties and Molecular Degradation of Schizostachyum Brachycladum Bamboo Cellulose Nanofibre in PLA-Chitosan Bionanocomposites

**DOI:** 10.3390/molecules26072008

**Published:** 2021-04-01

**Authors:** Samsul Rizal, N. I. Saharudin, N. G. Olaiya, H. P. S. Abdul Khalil, M. K. Mohamad Haafiz, Ikramullah Ikramullah, Umar Muksin, Funmilayo G. Olaiya, C. K. Abdullah, Esam Bashir Yahya

**Affiliations:** 1 Department of Mechanical Engineering, Universitas Syiah Kuala, Banda Aceh 23111, Indonesia; samsul_r@yahoo.com (S.R.); ikramullah@mhs.unsyiah.ac.id (I.I.); 2 School of Industrial Technology, Universiti Sains Malaysia, Penang 11800, Malaysia; mhaafiz@usm.my (M.K.M.H.); phunmieoseyemi@gmail.com (F.G.O.); ck_abdullah@usm.my (C.K.A.); essam912013@gmail.com (E.B.Y.); 3 Department of Physics, Universitas Syiah Kuala, Banda Aceh 23111, Indonesia; muksin.umar@unsyiah.ac.id

**Keywords:** molecular degradation, miscibility, reinforcement, sustainable, biocomposite

## Abstract

The degradation and mechanical properties of potential polymeric materials used for green manufacturing are significant determinants. In this study, cellulose nanofibre was prepared from Schizostachyum brachycladum bamboo and used as reinforcement in the PLA/chitosan matrix using melt extrusion and compression moulding method. The cellulose nanofibre(CNF) was isolated using supercritical carbon dioxide and high-pressure homogenisation. The isolated CNF was characterised with transmission electron microscopy (TEM), FT-IR, zeta potential and particle size analysis. The mechanical, physical, and degradation properties of the resulting biocomposite were studied with moisture content, density, thickness swelling, tensile, flexural, scanning electron microscopy, thermogravimetry, and biodegradability analysis. The TEM, FT-IR, and particle size results showed successful isolation of cellulose nanofibre using this method. The result showed that the physical, mechanical, and degradation properties of PLA/chitosan/CNF biocomposite were significantly enhanced with cellulose nanofibre. The density, thickness swelling, and moisture content increased with the addition of CNF. Also, tensile strength and modulus; flexural strength and modulus increased; while the elongation reduced. The carbon residue from the thermal degradation and the glass transition temperature of the PLA/chitosan/CNF biocomposite was observed to increase with the addition of CNF. The result showed that the biocomposite has potential for green and sustainable industrial application.

## 1. Introduction

Biopolymers are naturally occurring polymers with biodegradable properties [1]. Among the most abundant biopolymers are cellulose, chitosan, and starch. Biopolymers are extracted from plant or animal sources and often biocompatible with human systems. Biopolymer has been proposed as a possible replacement for synthetic polymers [2]. Biopolymers are more economical compared to synthetic polymers because they naturally exist. Most biopolymers are non-toxic, and these properties make them suitable for packaging application [3].

Several material types of research have been conducted on synthetic polymers for enhanced bio and thermal degradation [4]. Synthetic polymers such as polyethene, ABS, PVC have been blended with biopolymers for improved properties [5]. Further research on the use of biopolymer blends for packaging material showed poor mechanical properties. Biopolymers such as polylactic acid, chitosan, cellulose have been reported to have low mechanical properties [6]. This has discouraged their use for many applications such as automobile parts and aerospace. The mechanical properties of the proposed biopolymer have been of significant concern to researchers.

Polylactic acid (PLA) is one of the most researched biopolymers for composite film development [7]. PLA is in the focus of several researchers because of its potential properties, like synthetic ones. Apart from this, PLA is 100% biodegradable, as well as compostable [8]. PLA is prepared from natural renewable sources and processable on many plastic manufacturing machines conventionally designed for synthetic polymers. PLA is produced on an industrial scale from lactic acid condensation polymerisation [9,10]. Among biopolymers, PLA has higher thermal stability, making it suitable for automobile and packaging applications that require long-term use. As it is common to most biopolymers, PLA has low mechanical properties, and several types of research are being conducted to enhance its mechanical properties [11]. 

The use of nanoparticles as reinforcement for materials has restarted research on biopolymers for packaging [12]. Nanoparticles of cellulose and chitosan have been reported to significantly enhance many biopolymers’ mechanical properties [13]. The isolation of cellulose nanofibre from cellulose has been done using chemical and mechanical methods. Cellulose nanofibre has been used as reinforcement in most biopolymers for different applications. Research on polylactic acid and PBS has been on the front of material research for packaging application [13,14].

Chitosan has been blended with PLA to enhance its mechanical properties [15]. Chitosan is abundantly available in crustacea and has variously modified forms. Chitosan is a biodegradable and non-toxic biopolymer and has been used as a food supplement. Previous research on PLA/chitin biocomposite has reported no agglomeration between PLA and chitin blends [16,17]. However, chitosan also contains a hydroxyl group, which makes it behave like an amphiphilic polymer. The amphiphilic properties of chitosan have been reported in previous literature [18].

Cellulose nanofibre has been used as reinforcement of biopolymer for enhanced mechanical properties [7,19]. Cellulose nanofibre can be isolated from plant, animal and biomass [20,21,22]. However, it has been majorly isolated from wood-based plants. Recently studies have reported the isolation of cellulose nanofibre from bamboo species. Several isolation methods have been used to isolate cellulose, such as hydrolysis, mechanical and enzymatic methods [23]. Some studies have reported a combination of these methods to enhance the nanofibre yield [20]. In this study, Schizostachyum Brachycladum bamboo was selected because of its high aspect ratio, modulus of rupture, modulus of elasticity properties, as reported by Siam et al. [24]. Also, a combined chlorine-free bleaching, supercritical and high-pressure homogenisation was used to reduce toxicity from chlorine-based chemicals [25] and enhanced the yield of cellulose nanofibre [26].

Cellulose nanofibre has been used as reinforcement in PLA. It possesses a good strength enhancement to other biopolymers [7]. PLA-cellulose nanofibre biocomposite film properties have been researched with several agglomeration reports because of the difference in PLA and CNF [27,28,29]. CNF is hydrophilic, while PLA is hydrophobic. This nature reduces the miscibility between the two biopolymers. Chitosan is similar to cellulose in its chemical structure [30]. Chitosan and cellulose have been reported to have a similar molecular structure [31]. Their chemical structure similarity enhances cellulose and chitosan’s miscibility [32] and the possible hydrogen bonding formation. In this study, the miscibility between PLA and CNF was enhanced with chitosan as a compatibiliser. An extensive study on degradation properties and other functional properties of PLA/chitosan/CNF biocomposite was conducted. The combine properties of PLA/chitin/CNF bionanocomposite has not been fully researched for potential application.

## 2. Results and Discussion

### 2.1. Properties of Cellulose Nanofibre

**Figure 1:**
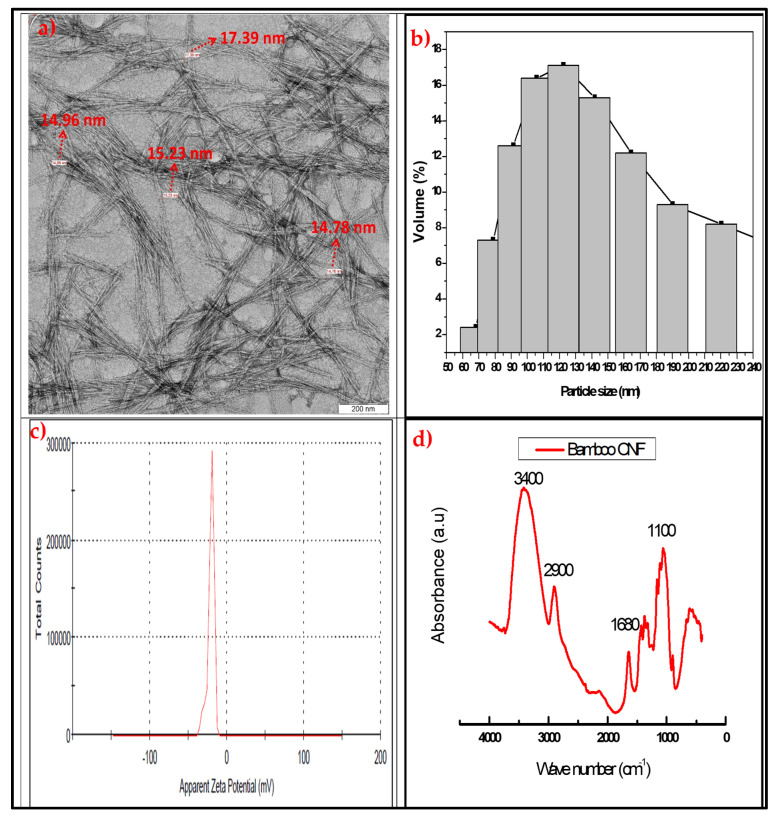
(**a**) TEM, (**b**) particle size analysis, (**c**) zeta potential, and (**d**) FT-IR analysis of *Schizostachyum brachycladum bamboo* cellulose nanofibre.

The properties of isolated cellulose nanofibres were obtained using transmission electron microscopy (TEM), particle size analysis, zeta potential measurement, and FT-IR functional group analysis. Figure 1a–d present the properties of isolated CNFs using combined supercritical carbon dioxide and high-pressure homogenisation. Figure 1a showed the transmission electron microscopy image of the CNF with the fibre diameter sizes as measured with the TEM software. The fibre length and diameter are nanosized as low as 14.98 nm. The fibres are broken and scattered on top of each other. The fibre formed an interlocking network of nanofibre strands. The result of the fibre diameter sizes was further confirmed with particle size analysis. The result of the CNF particle size distribution is presented in Figure 1b in percentages of particle sizes. The result shows particle sizes range of 60 nm to 220 nm with a peak at 119 nm. These particle size measurements are three-dimensional as they could generally represent the fibre diameter and sizes. This range of values confirmed the formation of cellulose nanofibre using these methods. 

The zeta potential value of isolated CNF was done to measure its colloidal stability. The result of the zeta potential analysis is presented in Figure 1c, and it’s a potential range between 0 and 50 V. the high voltage is an indication of stable particle materials. These confirmed that the CNF isolated could not be reversed back to its raw material or change its properties. Even at high voltage [33]. This means that the isolation process produced a stable cellulose nanofibre. Also, the FT-IR functional group analysis was used to confirm the effect of the isolation process on the chemical structure of the CNF. The absorbance spectra versus wavenumber of the CNF are presented in Figure 1d. The FT-IR absorbance stretched band for hydroxyl (OH) was observed between 3200 to 3600 cm^−1^. This is expected as cellulose contains OH in its chemical structure. The bands at 2900 cm^−1^ are the methylene group stretches. Also, the band at 1500 to 2000 cm^−1^ is assigned to C-H, and that at 1050 cm^−1^ is C-O stretching. These functional group bonds represent cellulose nanofibre, as reported in previous studies [28,34]. The functional group analysis confirmed the successful isolation of CNF using combined supercritical carbon dioxide and high-pressure homogenisation [33].

### 2.2. Properties of PLA/Chitosan/CNF Biocomposite

#### 2.2.1. Biodegradation Properties of PLA/Chitosan/CNF Biocomposite

**Figure 2:**
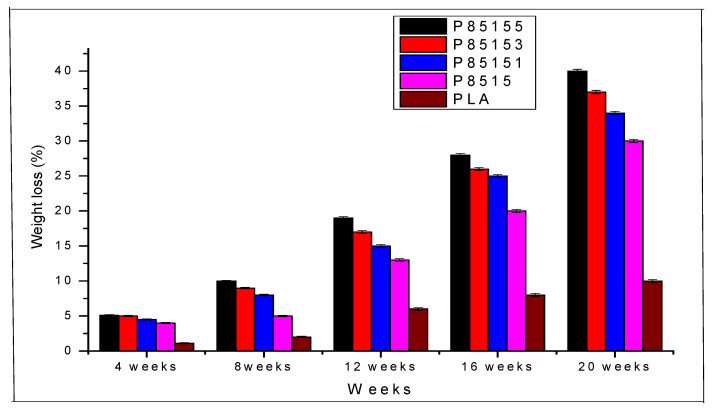
Biodegradation weight loss percentage of neat PLA, P8515, P85151, P85153, and P85155.

The degradation properties of the neat PLA and biocomposite were studied with soil biodegradability analysis. The biodegradation of the neat PLA, PLA/chitosan, and PLA/chitosan/CNF are shown in Figure 2. The sample images and weight were taken at intervals to measure the weight and observe the degradation process. After 75 days, the samples were observed to change in colouration [35,36].

The soil burial test was conducted for 150 days, and the change in weight of the biocomposite was recorded every month. Environmental degradation involves hydrolysis, microbial and enzymatic. The microorganisms (Nematodes) degradation will not begin until the hydrolytic process is complete [37]. The hydrolysis process breaks the ester bond, which the microorganism cannot do, into lower molecular weight (oligomer and monomers). This is accounted for the delay in the biodegradation process for about 45 days [37] at room temperature. PLA, chitosan, and CNF are naturally biodegradable, so the biocomposite is expected to degrade. However, the percentage composition of each biocomposite determines the rate. The weight loss percentages of neat PLA, PLA/chitin, and PLA/chitosan/CNF are plotted in Figure 2. 

The percentage of weight loss is observed to be higher with the addition of CNF and chitosan. The neat PLA showed 1.15 ± 0.11%, 2.21 ± 0.2%, 6.13 ± 0.2%, 8.03 ± 0.2%, and 10.10 ± 0.12% weight loss for the first, second, third, fourth and fifth month respectively. The degradation rate of neat PLA is not sporadic due to its hydrophobic nature. On the other hand, PLA/chitosan (P8515) is seen to show a higher degradation percentage of 2.10 ± 0.12%, 4.02 ± 0.13%, 10.11 ± 0.09%, 14.2 ± 0.10%, and 17.18 ± 0.14% for the first, second, third, fourth and fifth month compared to the neat PLA. The PLA/chitosan/CNF biocomposites showed an enhanced degradation rate than the neat PLA and PLA/chitosan. The degradation rate is enhanced with the addition of chitosan (15%). As observed from the degradation graph, the weight loss (%) of P85151 biocomposite was 30.04 ± 0.18%, increased to 34.64 ± 0.16% for P85153, increased to 40.28 ± 0.19% for P85155. Overall, neat PLA has the lowest percentage of weight loss while P85155 has the highest. The increased biodegradability is CNF, which is hydrophilic, and the biodegradable chitosan properties, which is easily degradable compared to PLA [38]. The soil burial test showed that the biocomposite fabricated is naturally degradable even though it has resistance to microbial attack due to the domination hydrophobic nature of PLA [39].

**Figure 3:**
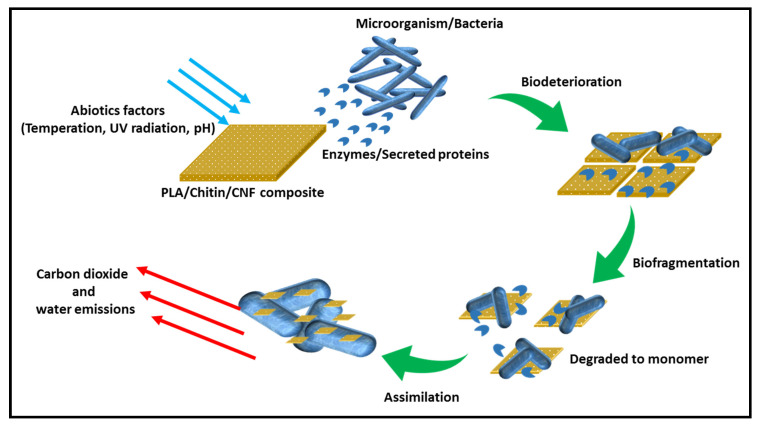
Schematic diagram of molecular enzymatic degradation process in soil burial test.

Soil burial degradation is both molecular, microbial and enzymatic. PLA degradation has been reported mainly due to phylogenetic Pseudonocardiaceae family such as Kibdelosporangium, Amycolatopsis, etc. The previous review on the degradation of PLA showed that it occurs in different ways, such as hydrolysis (molecular), microbial, thermal photodegradation, and enzymatic. However, the degradation studies of PLA in the soil mainly combine enzymatic and microbial [11]. PLA has a high tendency to degrade under soil since it contains different types of bacterial. However, as observed in these studies, the degradation of PLA took a long time because microbial action does not occur until after hydrolysis [11]. The schematic diagram of the degradation of the biocomposite is shown in Figure 3.

Furthermore, the presence of cellulosic material improved degradation. The biodegradable properties of PLA/chitosan/CNF were observed to increase with the addition of CNF. This probably showed that more microorganism was stimulated with increased CNF reported in the literature [36,40]. The biocomposite degradation probably occurs at the main chains and side chains, common to most biodegradable polyester [41]. The biodegradable polymer’s factors are the molecular weight distribution of constituent polymers, crystallinity, surface condition (hydrophobic or hydrophilic), and modulus of elasticity [36]. Based on a previous study on polylactic acid degradability [39], the biocomposite is first hydrolysed to lower molecular weight and mineralised by pseudonocardiaceae family microorganisms present in composite soil to carbon dioxide and water [11]. The degradation of polylactic-based biocomposite often takes longer than other polymeric materials because the initial degradation process is very slow and takes several weeks before starting in the compost soil. This was observed in the weight degradation plot (Figure 2 in this study [42]. PLA-based biocomposite can be hydrolysed into smaller oligomers, monomers, and dimers only after 60 days (at 60 °C) [11,37]. However, this process was observed to continue in this study even after 75 days (at room temperature). After which, it starts to chip off at the sides, degrading to carbon dioxide and water as seen in the degradation (Table 1) [36,37].

#### 2.2.2. Thermal and Mechanical Properties of PLA/Chitosan/CNF Biocomposite

**Figure 4:**
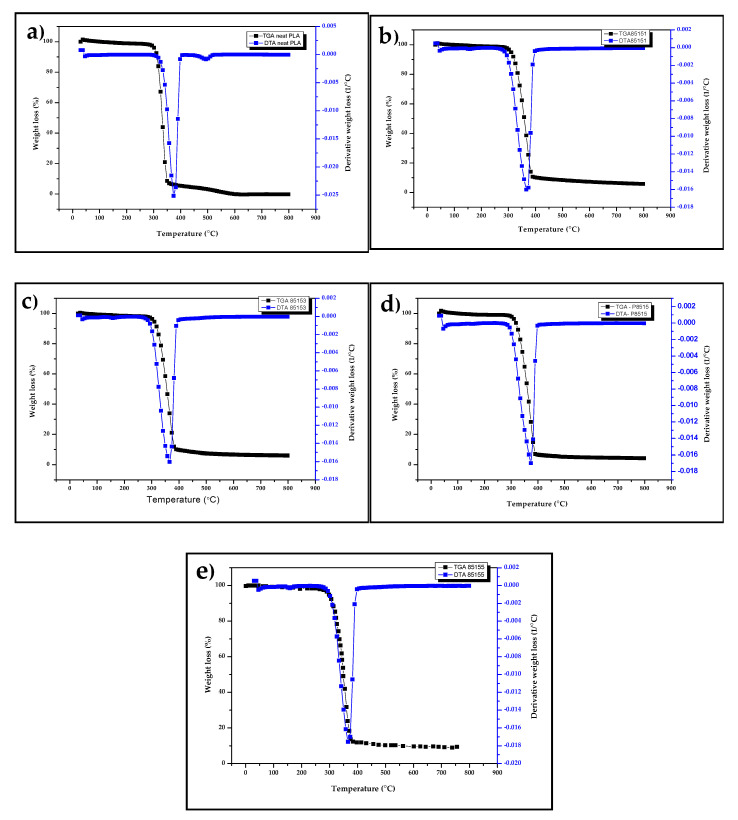
Thermogravimetry analysis (TGA) and derivative thermogravimetric (DTG) for (**a**) neat PLA, (**b**) P8515, (**c**) P85151, (**d**) P85153, and (**e**) P85155 biocomposites.

The thermal properties analysed with thermogravimetric analysis (TGA) and derivative thermogravimetric analysis (DTG) are shown in Figure 4a–e for neat PLA, PLA/chitosan, and PLA/chitosan/CNF biocomposite. The TGA result showed single degradation starting at onset temperature 276 °C, 280 °C, 284 °C, 290 °C, 298 °C for P85155, P85153, P85151, P8515, and PLA, respectively. The onset temperature value showed that chitosan and CNF significantly affect the biocomposites’ thermal degradation properties. The reduction in the onset temperature showed the possibility of degradability enhancement of PLA with chitosan and CNF [28]. Previous studies on PLA/chitosan and PLA/chitosan showed a similar trend attributed to natural fibre reinforcement’s biodegradable nature. Furthermore, the carbon residue percentage in the TGA curve increases with chitosan and chitosan compared with the neat PLA. Natural biopolymers like cellulose and chitosan have been reported to cause an increase in the carbon residue of biocomposite [30,34,43].

A similar trend was observed with the DTA graph’s peak temperature (Figure 4a–e). The result of DTA showed peak temperatures at 380 °C, 375 °C, 372 °C, 370 °C, and 367 °C for PLA, P8515, P85151, P85153, and P85155 respectively. The DTA curve’s peak value is often a reference to the maximum temperature where the properties of the material remain unchanged. Above this temperature, the material properties are not stable [43]. The thermal degradable properties of chitosan and CNF has a combined effect on the degradability of the biocomposite. A review of natural fibre reinforced in polymer composite reported that the use of these fibres in non-degradable polymer matrix enhanced their thermal degradability from the TGA studies [44,45]. The previous result on PLA/chitosan and PLA/CNF biocomposites showed a similar thermal degradation trend [36,46]. 

**Figure 5:**
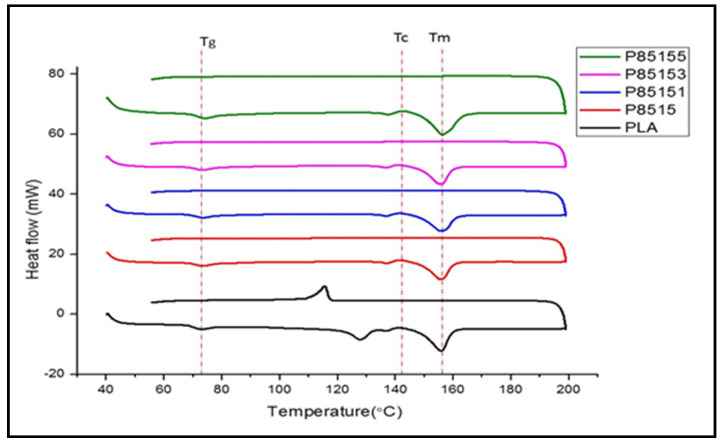
Differential scanning calorimetry for neat PLA, P8515, P85151, P85153, and P85155 biocomposites.

Further study on the thermal properties of biocomposite was conducted using differential scanning calorimetry. The result of heat flow with temperature change for heating and cooling curve of neat PLA, PLA/chitosan, and PLA/chitosan/CNF biocomposite is presented in Figure 5. The glass transition temperature (T_g_), crystallinity temperature (T_c_), and melting temperature (T_m_) of the neat PLA are shown in Figure 5 and the actual values presented in Table 2. 

The T_g_, T_c_, and T_m_ values were observed to increase with CNF and chitosan compared with neat PLA. The P8515 biocomposite transition temperature increase compared to neat PLA. The DSC result supported the fact that CNF has more crystalline parts, which enhanced its reinforcement effect. The increase in PLA T_g_ with chitosan’s addition is probably due to enhanced miscibility due to their hydrophobic nature. The T_g_, T_c_, and T_m_ values of PLA/chitosan increased due to interaction (physical or chemical) between the PLA and chitosan. The T_g_, T_c_, and T_m_ of PLA/chitosan/CNF (P85151, P85153 and P85155) generally increased compared with neat PLA but reduced compared with PLA/chitosan.

Furthermore, the transition temperatures were enhanced more with chitosan addition than CNF, probably due to the higher percentage of chitosan present [30]. However, the percentage of CNF was limited based on previous studies of agglomeration with PLA above 5%. The reinforcement ability of CNF contributed significantly to T_g_, T_c_, and T_m_’s enhancement, dependent on its nanosize, crystallinity, and bonding [30]. A similar result to this study was observed in previous studies’ thermal and mechanical tests on PLA [47] embedded with chitosan and CNF. In previous studies, this trend was attributed to the crystallinity of CNF, which required higher energy to break [28,30].

**Figure 6:**
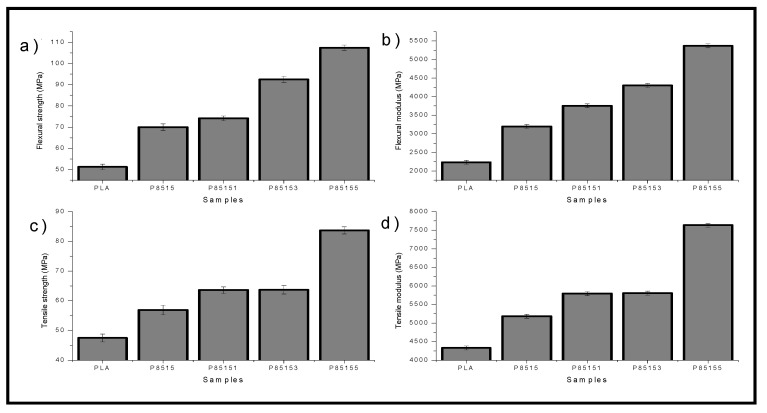
Mechanical properties (**a**) Flexural strength, (**b**) Flexural modulus, (**c**) Tensile strength, (**d**) Tensile modulus for neat PLA, P8515, P85151, P85153, and P85155 biocomposites.

The result of the mechanical (i.e., tensile and flexural) properties characterisation of neat PLA, P8515, P85151, P85153, and P85155 biocomposites is presented in Figure 6a–d.

The tensile properties of the neat PLA, P8515, P85151, P85153, and P85155 biocomposites are shown in Figure 6a,c. The tensile strength (Figure 6a) of the biocomposite increase with the addition of chitosan and CNF. The tensile strength values increased from 65 MPa for neat PLA to 89 MPa for 5% CNF (P85155) biocomposite. The result showed that the biocomposite’s tensile strength is further enhanced with the addition of CNF to the PLA/chitosan matrix, which justifies the reinforcement. The increase in tensile strength is probably due to the interaction (physical or chemical) and compatibility between the three polymeric materials [30]. The tensile strength result is an indication of enhanced miscibility. The inclusion of the reinforcement results in an even distribution of stress across the biocomposites.

Similarly, the tensile modulus (Figure 6c) of neat PLA and biocomposites increased with cellulose nanofibre addition. The tensile modulus increased significantly above 50% compared to the neat PLA. The modulus value range from 2500 MPa for neat PLA to 8500 MPa at 5% CNF (P85155). The incorporation of reinforcement in the PLA/chitosan matrix resulted in enhancing the material’s toughness and reduced the brittleness of PLA [48]. The addition of CNF increased the internal bonding of the polymeric material, which increased the modulus. The tensile properties of potential biopolymer composite for industrial applications are very significant [29]. The potential of using this PLA/chitosan/CNF in an application such as automobile dashboard, packaging, and biomedical parts is enhanced based on the result from this study [48].

The flexural properties of the biocomposite were analysed to determine its resistance to bending moment (rate of deflection). The flexural properties are very important for the application of the material in engineering. The result of the flexural strength and modulus is plotted in Figure 6b,d. The flexural strength (Figure 6b) of the biocomposite was observed to increase with the addition of CNF.PLA generally is brittle. The flexural strength result showed that the neat PLA’s flexibility is enhanced with the addition of CNF as reinforcement. The result observed that the 5% CNF (P85155) biocomposite had the highest flexural strength and the neat PLA the lowest. The result also showed that the flexural strength of the neat PLA is enhanced with chitosan. This is in accordance with the previous work of Nasrin et al. [17]. Similar studies on the flexural properties of PLA/CNF confirmed that the addition of cellulose nanofibre enhanced the flexural strength of PLA [30,49]. 

A similar trend was observed due to the flexural modulus of neat PLA, P8515, P85151, P85153, and P85155 biocomposite. The flexural strength range from 70 MPa to 91 MPa for neat PLA and P85155 biocomposite, respectively. The flexural modulus (Figure 6d) of neat PLA and biocomposite showed enhancement with cellulose nanofibre addition. The flexural properties have been reported to reduce in previous studies due to void and cracks in the biocomposite’s internal structure [19]. However, in this study, the flexural strength is observed to consistently increase with CNF addition, probably due to compatibility between the polymer constituents. The nanosize reinforcement has a high surface area that filled the voids [50]. The CNF nanoparticles contributed to enhancing PLA mechanical properties (flexural properties) because it has a high surface area [34].

Furthermore, the CNF nanoparticles have been reported to have bonding interaction with PLA and chitosan [30]. The reinforcement ability of CNF has been attributed to its nanosize and crystallinity [30]. The interfacial interaction of CNF results in reduced stress in the structural arrangement of the biocomposite. Flexural strength measures the material’s resistance to bending, while tensile strength measures its resistance to tension [51]. The flexural strength in Figure 6a is observed to be lesser than the tensile strength. Previous report data of polymer showed that the flexural strength is often higher than the tensile strength, which supports the validity of the mechanical properties observed in this study [30,49]. Furthermore, the flexural modulus was lower than the tensile modulus even though they have similar formula due to the reduced crossectional area of the dumbbell-shaped tensile samples [52]. 

**Figure 7:**
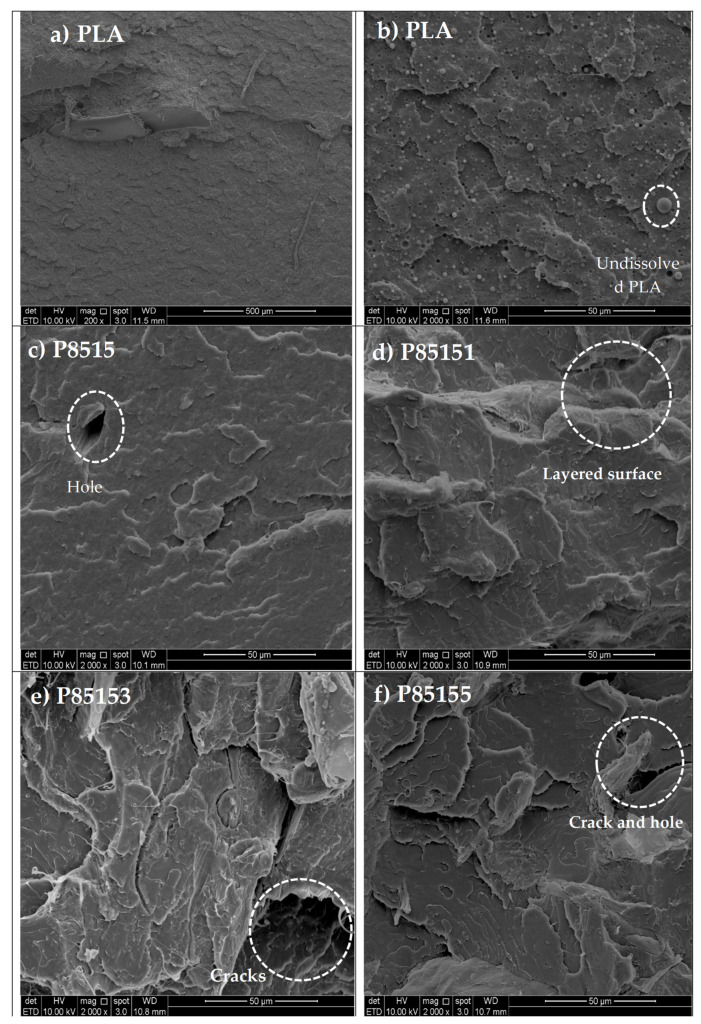
Tensile fractured surface SEM images for neat PLA, P8515, P85151, P85153, and P85155 biocomposites.

**Figure 8:**
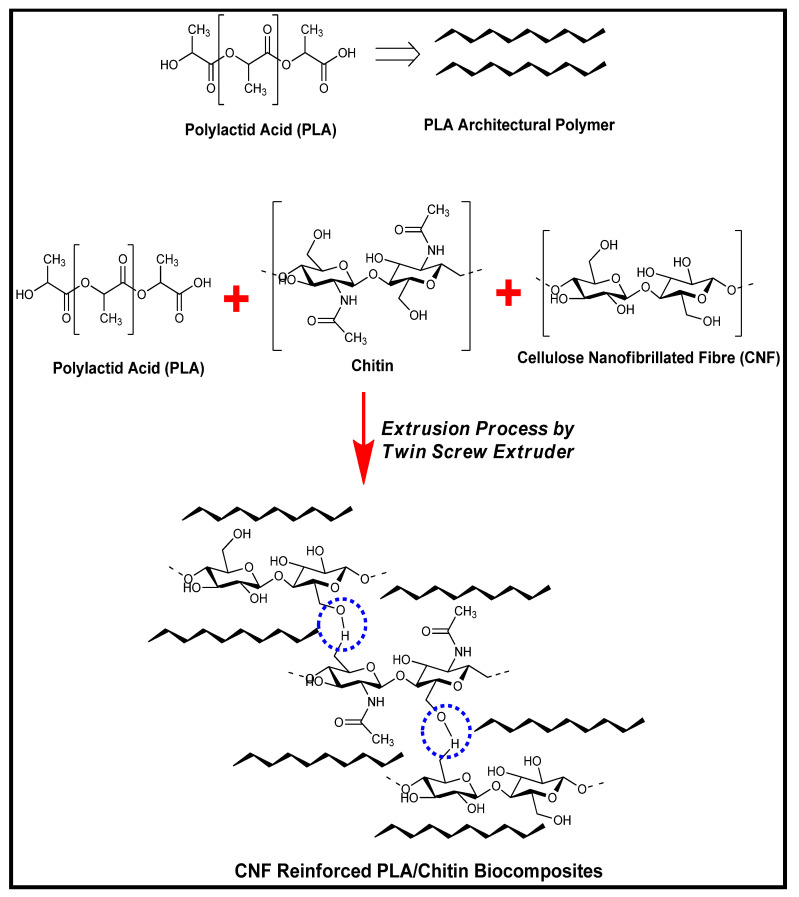
Schematic diagram of Possible chemical interaction between PLA, chitosan, and CNF

The fractured surface scanning electron microscope (SEM) of neat PLA, P8515, P85151, P85153, and P85155 biocomposites is shown in Figure 7a–f. The SEM images of the neat PLA is presented in Figure 8a–b at different magnification. The FESEM micrograph images in Figure 7 showed a distinct fracture image of neat PLA and biocomposite surface. 

The fibre reinforced biocomposite was easily identified in the micrograph with the increased presence of the CNF nanoparticles. Figure 7d–f (PLA/chitosan/CNF biocomposite) showed rough surface topography with the addition of chitosan and CNF compared to the neat PLA. The micrograph also reflected a compacted surface with a small void. Furthermore, the fracture surface showed no agglomeration or segregation of the fibre across it compared with neat PLA. Previous studies reported agglomeration in the morphology of PLA/CNF [28,34]. The layers of ridges with no aggregation on the fractured surface reflect enhanced miscibility between the polymers [53]. This can be explained with chitosan, which serves as a bridge between the hydrophilic CNF and hydrophobic PLA. Based on previous studies, chitosan showed uniform miscibility with PLA and CNF without agglomeration [30]. The enhanced miscibility increased the resistance of the biocomposite to tensile and bending forces compared to PLA. The polymer mix’s interfacial interaction probably enhanced uniform stress distribution when tensile, and bending forces were applied during the test [28]. The blend of PLA/chitosan/CNF showed better mechanical properties than those reported in previous studies on PLA/chitosan and PLA/chitosan biocomposites [15,16,17]. The SEM images also show that the forces (tensile and bending) did not cause disruption in the internal structure of the biocomposite but rather separated the plies into segments [34,43]. These micrograph images confirmed the high tensile and flexural properties of the biocomposite. Figure 8 showed the chemical reaction between the three polymeric material.

#### 2.2.3. Physical Properties of PLA/Chitosan/CNF Biocomposite

**Figure 9:**
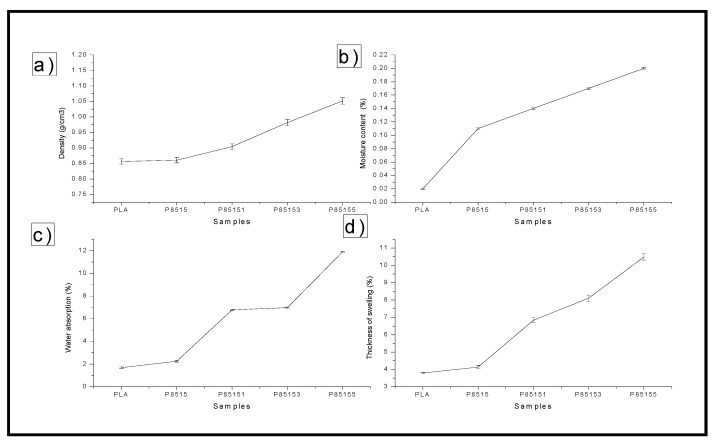
Physical properties (**a**) Density, (**b**) Moisture content, (**c**) water absorption, (**d**) Thickness swelling for neat PLA, P8515, P85151, P85153, and P85155 biocomposites.

The result of the physical properties (i.e., moisture content, density, thickness swelling, and water absorption) characterisation of neat PLA, P8515, P85151, P85153, and P85155 biocomposites is presented in Figure 9a–d.

**Figure 10:**
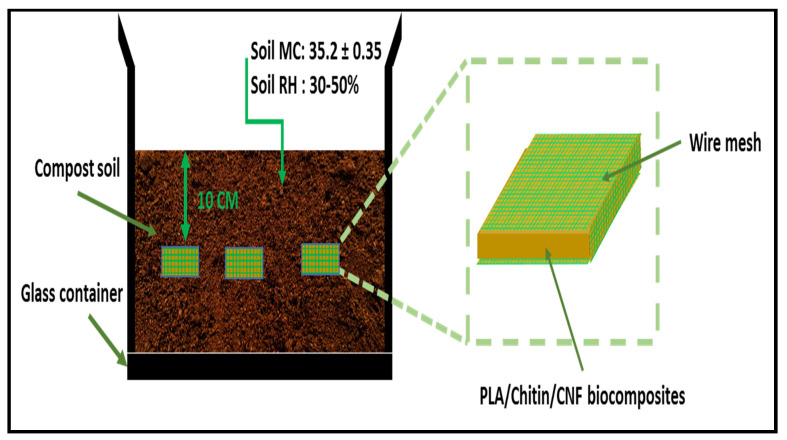
Schematic drawing of soil burial test set up for molecular biodegradation of neat PLA and biocomposite.

The neat PLA’s density value, P8515, P85151, P85153, and P85155, are plotted in Figure 10a. The neat PLA has the lowest density while the biocomposite with 5% CNF had the highest. Similarly, the chitosan addition to the matrix enhanced the PLA density. The result showed that the loading of CNF increases the density of the material. The increase in density value with the addition of CNF is probably due to the fibre’s nanosize [53]. Nanoparticles probably filled up possible void or hollow space in the biocomposite bundles, resulting in compacted material. Mathematically, density is calculated from mass or weight per unit volume. This means that the biocomposite mass increases with the addition of CNF while still occupying the same volume resulting in an increased density value [53].

The moisture content for neat PLA, PLA/chitosan biocomposite, and PLA/chitosan/CNF biocomposite was plotted in Figure 9b. The graph showed that the samples’ moisture content varies between 0.02% to 0.20%. The neat PLA was observed with the lowest moisture content. In comparison, the biocomposite with 5% cellulose nanofibre has the highest (0.2%) moisture content. The moisture content increases with the addition of chitosan and cellulose nanofibre compared with that of neat PLA. The lowered value of the neat PLA’s moisture content is probably due to the hydrophobic nature of PLA. The addition of chitosan to the neat PLA was observed to increase its moisture content slightly, probably due to the hydroxyl functional group present in chitosan apart from the amine group. However, the addition of CNF has a greater effect on the biocomposite’s moisture content because of the hydrophilic nature of the CNF [28]. The CNF has higher water content, and its addition to PLA/chitosan increases its moisture content. The difference in the moisture content between PLA/chitosan and PLA/chitosan/CNF is explained by introducing more hydroxyl groups with high CNF content. A previous study on the moisture content of PLA/chitosan and PLA/chitosan showed a similar trend. The moisture content from the study of PLA/chitosan composite by Nasrin et al. [17] showed an increase with the addition of a higher percentage of chitosan. Also, similar studies confirmed an increase in PLA/chitosan composite’s moisture content compared with the neat PLA [15,54]. They explained that it is probably due to space between the PLA and chitosan’s molecular arrangement at a higher percentage. Studies on PLA/chitosan showed that the addition of CNF added to the moisture content because of the water-containing cellulose ability [28,30,34].

When immersed in distilled water for 24 h, the biocomposites’ percentage absorption is plotted in Figure 9c. The biocomposite’s water absorption properties are very important because they determine the environment where the material can be used and its dimensional stability. A biocomposite with high water absorption may cause a weakening of its internal bonding, reducing its mechanical strength. Fibre loading is one of the parameters that significantly affect the water absorption properties of biocomposite [29]. The water absorption percentage was observed to increase when compared with the neat PLA significantly. The water absorption value increased with the addition of chitosan and CNF. The water absorption values between neat PLA and PLA/chitosan seem to be close despite 15% of chitosan but significantly higher with CNF. This showed that the addition of CNF has a higher effect on water absorption properties than chitosan. This is probably due to the hydrophilic nature of CNF because of its crowded hydroxyl presence in its structure. Chitosan, on the other hand, is considered hydrophobic because it is not soluble in water. This difference in chitosan and CNF nature has a greater impact on the water absorption properties of the resulting PLA/chitosan/CNF. The water absorption value result showed that the properties depend on the fibre reinforcement’s nature and the reinforcement’s quantity in the PLA. This observation is similar to the previous studies on water absorption properties of PLA containing chitosan or CNF [29,30]. In these studies, it was reported that the nature of the filler or fibre reinforcement has a significant effect on the water absorption properties.

Furthermore, chitosan being hydrophobic, provide more water absorption stability to the biocomposite than the CNF. The increase in CNF in the biocomposite results in neat PLA’s ability to form hydrogen bonding with water [34]. This resulted in more water absorption of the biocomposite with the addition of CNF. However, the biocomposite water absorption properties showed characteristics of hydrophobic material.

The thickness swelling of neat PLA, PLA/chitosan, and PLA/chitosan/CNF biocomposites after immersion in water for 24 h is plotted in Figure 9d. The thickness of the PLA/chitosan and PLA/chitosan/CNF increased compared with that of the neat PLA. The trend of the thickness is similar to that of water absorption. The thickness of the swelling of the neat PLA did not significantly change due to its hydrophobic nature. The thickness of the PLA’s swelling increased with chitosan’s addition, as observed in the P8515 biocomposite. This is probably due to the space created by the presence of chitosan particles in the PLA matrix. Also, the thickness swelling of PLA/chitosan/CNF (i.e., P85151, P85153, and P85155) increased with the addition of CNF, which the hydrophilic nature of CNF can explain. Generally, this study’s thickness swelling depends on the polymer mix’s nature and the porosity (voids or space) between the polymer mix molecules [55,56]. The percentage thickness swelling value is quite low, which showed that the biocomposite has low porosity and is highly resistant to water [57]. 

## 3. Materials and Methods

### 3.1. Materials

The cellulose nanofibre was isolated from *Schizostachyum brachycladum bamboo*. Polylactic acid (4032D) was purchased from Sigma Aldrich, Malaysia, with 1.24 specific gravity, melt extrusion temperature of 55 °C to 60 °C, 53 MPa tensile strength, 60 MPa yield strength and tensile modulus of 3500 MPa, respectively. Also, deacetylated practical grade chitosan (90%) with a melting point of 102.5 °C and a density of 1 g/cm^3^ was purchased from Sigma Aldrich for this study.

### 3.2. Preparation of Cellulose Nanofibre and PLA/Chitosan/CNF Biocomposite (Composition Variation) 85% Series

The composition variation between PLA and chitosan was kept constant at 85:15 percentage ratio while the CNF was varied between 1%, 3%, and 5% according to previous literature [17,28]. Table 3 presents the composition variation used in this study. 

The isolation of cellulose from *Schizostachyum brachycladum bamboo* was done using a modified method of Atiqah et al., [33]. Raw *Schizostachyum brachycladum bamboo* culm was cut into small sizes (5–10 mm) and was digested with sodium hydroxide (NaOH) in a high-pressure digester for 4 h. The digested fibres were washed with water to remove excess NaOH and bleached with hydrogen peroxide (H_2_O_2_) and ozone (O_3_). The chlorine-free bleached fibre was washed with water to remove excess chemicals, and this was followed by acid hydrolysis using hydrogen tetraoxosulphate (VI) to obtain the cellulose. The cellulose was exposed to high-pressure supercritical CO_2_ for 4 h and high-pressure homogenisation for 24 h to obtain cellulose nanofibre [58]. The CNF was characterised with FT-IR analysis, TEM, zeta potential and particle size analysis. The FT-IR sample was prepared from the CNF powder by mixing it with potassium bromide (KBR) and pressed into the circular film. The film was placed in the FT-IR EFTEM Libra–Carl Zeiss, UK, and the transmittance spectrum of the material was obtained. The TEM samples were prepared by dissolving the 0.05 mg dried CNF in water. A drop of the samples was placed on copper foil and stained with acetone before placing it in the TEM Perkin-Elmer, PC1600, Winter Street, Waltham, MA, USA. The TEM images were obtained and the fibre diameter measured using TEM software using image J software as indicated on the TEM image. The fibre size was further confirmed using a particle size analyser (Zetasizer Ver. 6.11, Malvern, UK, and the fibre diametre percentage distribution in nanometre obtained. Aqueous suspension of 1% wt CNF in water was prepared and examined based on ISO 13320-1:1999 in the machine [59]. Also, the isolated CNF colloidal stability was measured with zeta potential analysis. The CNF zeta potential for stability was analysed with Zetasizer Ver. 6.11 (Malvern, UK). The CNF suspension was prepared using 0.01mg in 5ml of distilled water of refractive index 1.330 and sonicated for 10 min 6. The zeta potential was obtained in 0.1 mM KCl electrolyte using the machine. 

The isolated cellulose nanofibre was used as reinforcement in PLA/chitosan. PLA (85% wt.) was mixed with chitosan (15%) and reinforced with 1%, 3%, 5% of CNF using the HAAKE Rheomix OS Lab Mixers system. The polymer mix was extruded in a twin-screw extruder Thermo Electron Process 11 extruder (Thermo Fisher Scientific, Waltham, MA, USA) and pelletised Thermo Scientific Varicut Pelletizer 11M (Thermo Fisher Scientific, Waltham, MA, USA). The polymer mix was extruded at a temperature profile of 120–180 °C at a 100-rpm extrusion rate. The PLA/chitosan/CNF biocomposite pellets were poured into a rectangular mould and pressed into the plate with a carver compression moulding machine (model 3851-0) (Carver, Wabash, IN, USA) at 170 °C. The rectangular plate was cut into test samples and kept in a zip-lock bag. Five (5) replicates per sample composition was used for the characterization.

### 3.3. Characterisation of Neat PLA, PLA/Chitosan, and PLA/Chitosan/CNF

#### 3.3.1. Molecular Degradation Properties

Furthermore, the biodegradability properties of the material were obtained using a soil burial test. The samples (3 per composition) were cut into 2 cm × 2 cm and buried 10 cm below compost soil for 150 days, as shown in Figure 10. 

The neat PLA and biocomposites samples were preweighed before burial and after that weighed at the one-month interval to measure the degradation rate. The samples are taken out and washed with distilled water, dried in an oven for 24 h at 40 °C before weighed. The soil burial test was conducted based on previous studies and ISO 846 (Plastics-Evaluation of microorganisms’ action). The compost soil’s relative humidity is kept between 40–50% at room temperature, and the soil was constantly injected with distilled water to maintain its moisture condition for microbial activities. The percentage of weight loss is calculated from Equation (1).

(1) $$Weigth\;loss\;\left(\%\right)=\frac{W_{1}-W_{2}}{W_{1}}\times 100$$

#### 3.3.2. Thermal and Mechanical Properties Characterisation

The material’s thermal properties were obtained using PerkinElmer TG-IR-GCMS Interface Q500, TA Instruments (PerkinElmer Inc., Akron, OH, USA) at 20 °C/min for temperature interval of 40 °C to 800 °C. The thermogravimetric analysis (TGA) and derivative thermogravimetric (DTG) measurement of mass reduction with temperature were done for neat PLA and biocomposites. The TGA and DTG properties were measured to study the thermal degradation properties of the material. The differential scanning calorimetry analysis was done at a temperature of 50 to 200 °C, with a temperature increase rate of 20 °C/min. The transition temperature (T_g_), melting temperature (T_m_), and crystallisation temperature (T_c_) for each sample was obtained. The DSC was done with Perkin–Elmer differential scanning calorimetry (DSC) model 6 (Perkin–Elmer, Schwerzenbach, Switzerland) machine and mass analysed varies between 5 mg to 10 mg per test sample in powdered form.

The mechanical properties of the biocomposite were measure with tensile and flexural tests. The tensile properties of neat PLA, PLA/chitosan, and PLA/chitosan/CNF were measured using the Instron Universal testing MT1175 (Dia-Stron Instruments, Andover, UK) machine. The test was carried out at test standard ASTM D638 with dimensions 165 mm × 19 mm × 3 mm, 50 KN. The tensile strength and modulus values of neat PLA and biocomposite were recorded. The flexural test was conducted using ASTM D790 standard polymer composite testing. The samples were moulded into a rectangle of 200 mm × 12.7 mm × 3 mm and placed in the Instron universal testing MT1175 (Dia-Stron Instruments, Andover, UK) machine. A machine load capacity of 50 KN was applied at a rate of 2 mm/min. The flexural strength and modulus were obtained and analysed for neat PLA and biocomposites. The tensile fracture surface’s morphological properties were studied with scanning electron microscopy to study the three (3) polymeric material’s miscibility. The morphology study was conducted to explain further the values of the tensile strength and modulus obtained using FESEM EVO MA 10, Carl-ZEISS SMT, Oberkochen, Germany.

#### 3.3.3. Physical Properties Characterisation

The physical properties of the PLA/chitosan/CNF were studied with moisture content determination, water absorption, thickness swelling, and density measurement. The moisture content of the biopolymer composite was determined using ASTM D6980-17. standard. PLA/chitosan/CNF biocomposite was cut into 2 cm × 2 cm and preweighed before being oven-dried at 60 °C until a constant weight is achieved. The value of the final weight was obtained, and the moisture content was determined using Equation (2).

(2) $$Moisture\;content\;\left(\%\right)=\frac{W_{1}-W_{2}}{W_{1}}\times 100$$

where *W*_1_ and *W*_2_ are the initial and final weights of the samples.

Similarly, the percentage of thickness swelling was measured by pre-weighing 2 cm × 2 cm cut samples and immersing them in 500 mL distilled water. The final weight of the samples was taken after 24 h according to ASTM D570–98, and water absorption properties were determined using Equation (3)

(3) $$Water\;absorption\;\left(\%\right)=\frac{W_{2}-W_{1}}{W_{1}}\times 100$$

where *W*_1_ and *W*_2_ are the initial and final weights of the samples.

The thickness of the biocomposite’s swelling was calculated by measuring the water absorption samples’ initial and final thickness using a micrometre screw gauge. The value of the percentage thickness swelling was calculated with Equation (4)

(4) $$Thickness\;of\;swelling\;\left(\%\right)=\frac{t_{2}-t_{1}}{t_{1}}\times 100$$

where *t*_1_ and *t*_2_ are the initial and final thickness of the samples.

The biocomposite density was determined by measuring the mass (*m*) and thickness (*t*) of 2 cm × 2 cm cut samples. The density was calculated using Equation (5)

(5) $$Density\;\left(\rho \right)=\frac{m}{V}$$

where *V* = l × b × *t* is the volume of the cut samples.

## 4. Conclusions

Cellulose nanofibre was successfully isolated from *Schizostachyum brachycladum* bamboo stalk and used as reinforcement in PLA/chitosan biocomposite to enhance its physical, mechanical, and degradation properties. The properties of CNF isolated with combined supercritical carbon dioxide and high-pressure homogenisation were observed to show typical characteristics similar to previous studies. The PLA/chitosan/CNF was produced with melt extrusion and compression moulding technic and characterised. Physical properties such as density, moisture content, water absorption, and thickness swelling were observed to increase with the addition of CNF. Mechanical properties of the biocomposite increased significantly compared with the control samples. Furthermore, the thermal and biodegradation properties were improved with the addition of without reduction in the biocomposite’s mechanical strength. The biocomposite is suitable for green and sustainable material application in automobile parts, packaging, and other industrial use.

## Figures and Tables

**Table 1:** Biodegradation sample result of neat PLA and biocomposites.

Scheme 75.	Before Soil Burial	After 75 Days	After 150 Days
P85155	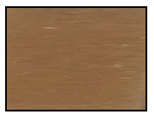	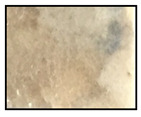	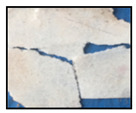
P85153	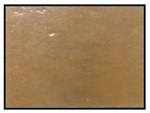	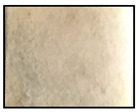	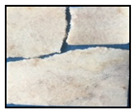
P85151	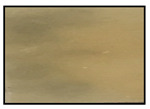	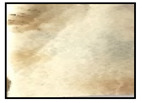	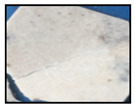
P8515	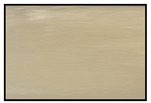	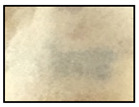	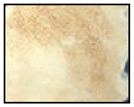
PLA	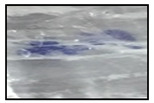	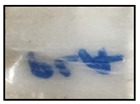	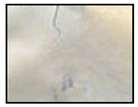

**Table 2:** T_on_ and T_peak_ of neat PLA, PLA/chitosan, and PLA/chitosan/CNF biocomposite.

Scheme.	Transition Temperature	Crystallisation		Melting	
	T_g_ (°C)	T_on_ (°C)	T_max_ (°C)	T_on_ (°C)	T_max_ (°C)
P85155	73.22	133.86	137.03	148.21	155.42
P85153	72.93	133.34	136.65	147.41	155.49
P85151	73.57	134.81	137.99	148.49	156.91
P8515	73.68	134.11	137.43	148.42	157.07
PLA	72.86	134.84	137.40	148.56	155.83

**Table 3:** Percentage composition variation.

Sample Name	Polylactic Acid (wt%)	Chitosan (wt%)	CNF (wt%)
P85155	85	15	5
P85153	85	15	3
P85151	85	15	1
P8515	85	15	0
PLA	100	0	0

Note: CNF (wt%) is with respect to the total weight of the polymer matrix.

## Data Availability

Not applicable.

## Sample Availability

Samples of the compounds are available from the authors.
